# Smart copper-doped clays in biomimetic microparticles for wound healing and infection control

**DOI:** 10.1016/j.mtbio.2024.101292

**Published:** 2024-10-09

**Authors:** Marco Ruggeri, Cristian Nomicisio, Christine Taviot-Guého, Barbara Vigani, Cinzia Boselli, Pietro Grisoli, Antonia Icaro Cornaglia, Eleonora Bianchi, César Viseras, Silvia Rossi, Giuseppina Sandri

**Affiliations:** a Department of Drug Sciences, University of Pavia, Viale Taramelli 12, 27100, Pavia, Italy; b Institut de Chimie de Clermont-Ferrand, Université Clermont-Auvergne, UMR CNRS 6296, 24 av Blaise Pascal, 63171, Aubière, France; c Department of Public Health, Experimental and Forensic Medicine, University of Pavia, via Forlanini 2, 27100, Pavia, Italy; d Department of Pharmacy and Pharmaceutical Technology, Faculty of Pharmacy, University of Granada, Campus of Cartuja, 18071, Granada, Spain

**Keywords:** Chronic wounds, Biomimetic scaffolds, Clays, Antimicrobial properties, Wound healing

## Abstract

Chronic wounds are non-healing lesions characterized by a high degree of inflammation, posing significant challenges in clinical management due to the increased risk of severe infection. This study focuses on developing a powder for cutaneous application to enhance the healing and prevent infections in chronic wounds. The smart nanocomposites-based biomimetic microparticles here developed combine the properties of chitosan and of clays and represent a significant innovation in the field of biomaterials for skin regeneration since they possess enhanced antimicrobial properties, are multi-functional scaffolds and promote cell proliferation, support tissue reconstruction by mimicking the natural extracellular matrix, and provide hemostatic properties to control bleeding during wound closure. The microparticles were made of chitosan and doped with clay minerals, specifically montmorillonite or layered double hydroxides, containing copper ions. The synergistic combination of biomimetic polymers and clays aims to regulate cellular responses, angiogenesis, and extracellular matrix (ECM) deposition, leveraging the bioactive properties of both components to promote wound healing. Montmorillonite and layered double hydroxides were enriched with copper ions through intercalation or coprecipitation methods, respectively. The water-insoluble microparticles were prepared using a chitosan derivative, chitosan carbamate, synthesized to obtain chitosan-based microparticles via spray-drying without crosslinkers. Physico-chemical characterization confirmed the successful doping of Cu-clay interaction products in the microparticles. In addition to enhanced cell proliferation and hemostatic properties, the presence of Cu-clays boosted the microparticles’ antibacterial properties. Encouraging preclinical *in vitro* and *in vivo* results suggest that these smart nanocomposite biomimetic microparticles doped with Cu-enriched clay minerals could be promising candidates for simultaneously enhancing healing and controlling infections in chronic wounds.

## Graphical abstract

**Figure:**
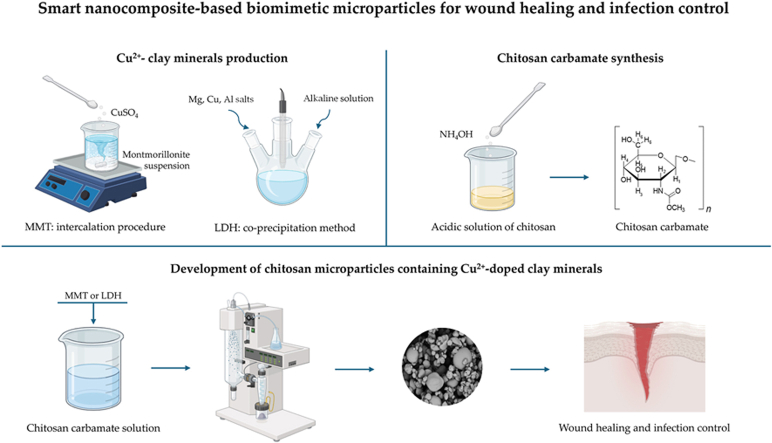

## 1 Introduction

Chronic wounds, characterized by impaired healing processes and prolonged inflammation, pose significant challenges in clinical management [1]. These wounds often lead to debilitating complications and to significant pain and discomfort, impairing mobility and reducing quality of life for patients. Chronic pain associated with wounds can lead to depression, anxiety, and sleep disturbances, which may exacerbate underlying health conditions and increase mortality risk [2,3]. In addition, persistent open wounds can provide a favorable environment for bacterial colonization and proliferation, which can lead to localized or systemic infections [4]. In severe cases, these infections can spread to surrounding tissues or enter the bloodstream causing sepsis, a life-threatening condition characterized by a systemic inflammatory response [5]. Sepsis can lead to multiple organ dysfunction syndrome and, if not promptly treated, may result in death [6].

In this context, clay minerals have been proposed as promising nanomaterials for designing and developing scaffolds intended for skin repair. Depending on their composition, clays are characterized by high aspect ratio, high surface area, ion exchange capacity, and tunable surface chemistry, which support their interactions with biological molecules, cells, and tissues. These interactions influence and enhance cell behavior, signaling pathways, and physiological responses [7,8]. Clays interact through various mechanisms, including adsorption, ion exchange, and surface-mediated reactions. They also possess antimicrobial properties, which disrupt microbial membranes and modulate bacterial growth and biofilm formation, reducing infection risks and increasing the efficacy of tissue engineering scaffolds [9,10].

Montmorillonite and layered double hydroxides are two prominent classes of layered clays with unique structures and properties, depending on the ions that constitute their compositions. Montmorillonite belongs to the smectite group of clay minerals and is primarily composed of aluminium and magnesium silicate layers [11]. These layers are held together by weak van der Waals forces, allowing them to swell and expand upon hydration [12,13]. The interlayer space in montmorillonite can accommodate various cations, such as sodium (Na^+^), calcium (Ca^2+^), and potassium (K^+^). Conversely, layered double hydroxides are composed of positively charged metal hydroxide layers and interlayer anions [14]. Their general formula is [M^2+^_1-x_M^3+^_x_(OH)_2_]^x +^ [X^n-^_m/x_∗nH_2_O], where M^2+^ and M^3+^ represent divalent and trivalent metal cations, respectively, such as magnesium (M^g2+^), aluminum (Al^3+^), zinc (Zn^2+^), and nickel (Ni^2+^) [15,16]. The interlayer anions can vary and include species such as carbonate (CO_3_^2−^), nitrate (NO_3_^−^), and chloride (Cl^−^).

Both montmorillonite and layered double hydroxides exhibit high ion exchange capacities of cations and anions present in surrounding solutions. Montmorillonite shows high resistance to degradation due to its mineralogical nature, while the degradation of layered double hydroxides depends on the interlayer anions and specific metal cations present [17]. Generally, acidic environments tend to disintegrate the hydroxide layer of layered double hydroxides and accelerate metal dissolution [18,19].

Recently, the synergistic combination of biomimetic polymers and clays has been shown to enhance mechanical strength and modulate cell responses to achieve wound healing [20,21]. Chitosan, a naturally occurring polysaccharide derived from chitin, is a biomimetic polymer that provides a suitable microenvironment for wound healing, mimicking the structural and biochemical cues of native tissues [22,23]. This polymer possesses ideal properties for biomedical applications, including biocompatibility, degradability, antibacterial, and hemostatic properties [24,25]. These features allow chitosan to effectively interact with biological tissues, making it a valuable candidate for wound healing and tissue engineering. However, a major challenge influencing the application of chitosan in these fields is its solubility behavior. Chitosan exhibits pH-dependent solubility: it is insoluble in alkaline conditions but becomes soluble in acidic solutions due to the protonation of its amino groups [26,27]. This pH-responsive solubility enables the controlled release of bioactive molecules from chitosan-based scaffolds in response to the physiological pH environment of wounds.

Given these premises, this work is aimed at the design and development of chitosan-based microparticles doped with montmorillonite or layered double hydroxides containing Cu^2+^ ions, as a powder for cutaneous application to enhance healing and prevent infections in chronic wounds. In particular, these clay minerals were selected as proliferation enhancers and were enriched with Cu^2+^ ions to ensure its controlled release for prolonged antimicrobial activity. To this end, Cu^2+^ was intercalated in montmorillonite, while Cu^2+^ was included in layered double hydroxides during synthesis through coprecipitation [15,28]. Chitosan carbamate, a derivative soluble in neutral environment, was used as a chitosan precursor since it can be processed without the need for acidic solvent, avoiding the degradation of layered double hydroxides. The clays were then mixed with the polymeric matrix and subjected to the spray-drying process, as a sustainable and scalable method. This process also allowed to convert chitosan carbamate into pristine chitosan, preventing its solubilization upon contact with the physiological aqueous environment, thus using a green process without chemicals and solvents. The chitosan-based microparticles doped with Cu-montmorillonite or Cu-layered double hydroxides were characterized using a multi-disciplinary approach: physico-chemical properties (morphology, size distribution, solid state, and thermal profiles) and preclinical properties (*in vitro* degradation, copper release and antimicrobial activity, cytocompatibility, *in vitro* wound healing assay, and *in vivo* safety and efficacy on a murine model) were assessed.

The innovative nature of this research lies in the use of clay minerals as enhancers of the antimicrobial activity due to the inclusion of copper, a bioactive ion, as an attractive alternative to fight antimicrobial resistance. Moreover, in this research, the distinctive features of clay minerals, as booster of cell proliferation and tissue regeneration, will be investigated aiming at improving the performance of the chitosan matrix. The comparison of the effects of natural and synthetic clay minerals as scaffold doping should demonstrate how their peculiar properties could affect the system. The research is also focused on the scalability of both the production of copper-based clays and the manufacturing of the microparticles as sequential processes.

## 2 Materials and methods

### 2.1 Materials

Mg(NO_3_)_2_∗6H_2_O (pur. 99 %, Sigma-Aldrich, France), Cu(NO_3_)_2_∗3H_2_O (pur. 99 %, Sigma-Aldrich, France), Al(NO_3_)_3_∗9H2O (pur. 99 %, Sigma-Aldrich, France), 25 % NH_4_OH solution (Sigma-Aldrich, France) were used for layered double hydroxides-Cu^2+^ synthesis. A pharmaceutical grade montmorillonite (particle size: 1352 nm (±17), polydispersity index: 0.696 (±0.184), Veegum® HS, Vanderbilt, USA) and CuSO_4_ (Sigma-Aldrich, Italy) were used for montmorillonite-Cu^2+^ intercalation. Chitosan (CHS, β-(1–4)-linked D-glucosamine and N-acetyl-D-glucosamine, low MW 251 kDa, deacetylation degree 98 %, ChitoClear, Siiiglufjordur-Iceland) and ammonium bicarbonate (Sigma-Aldrich, Italy) were used for chitosan carbamate synthesis.

### 2.2 Methods

#### 2.2.1 Preparation of MMT and LDH

The intercalation method was used to obtain montmorillonite-Cu^2+^ intercalation complex (MMT) [28]. 1.5 g of montmorillonite was added to 40 mL of distilled water and kept under magnetic stirring for 1 h. Then, 80 ml of CuSO_4_ solution (6.25 mg/ml) were added to the montmorillonite suspension and stirred for 24 h. Finally, the suspension was centrifuged at 9000 rpm for 45 min and the residue obtained was dried in an oven and sieved (150 μm mesh).

Layered double hydroxides-Cu^2+^ was prepared using the coprecipitation method (LDH) [15]. Briefly, 20 mL of an aqueous solution of nitrate salts containing the cations (Mg, Al, Cu, at concentration of 0,075 M) was added dropwise at a constant flow rate (0.67 ml/min) by means of a peristaltic pump in a reactor filled with 100 ml of water at room temperature, under N_2_ atmosphere and upon magnetic stirring. The pH of the reaction was maintained constant at 9 and controlled by the addition of a 1M NH_4_OH solution. The total addition time was set to 30 min and the resulting precipitate was aged for 1h. Then, the synthesized LDH were recovered through centrifugation (4500 rpm for 10 min) and stored as a slurry, with no intermediate washing steps.

#### 2.2.2 Physico-chemical characterization of MMT and LDH

MMT and LDH were sputtered with graphite and imaged with SEM (Tescan, Mira3XMU, Czech Republic) at 9–15 kV voltage, under high vacuum and at room temperature.

The Cu contents of MMT and LDH were assessed by means of ICP-OES (Agilent 5800, Agilent Technologies). As for MMT, Cu was indirectly determined by measuring the ion amount in the supernatant after centrifugation, and Cu content was calculated as the difference between the initial concentration and the concentration in the supernatants. As for LDH, a fixed amount of clay was dissolved in concentrated nitric acid, digested at 230 °C for 30 min and total Cu content was determined.

X-ray powder diffraction (XRPD) was performed by means of a diffractometer (X-Pert Pro® Marvel Panalytical, Madrid, Spain) with the CuKα radiation (*λ* = 1.5405 Å) in a 2θ angle range of 4°–70°.

Thermal analysis (TGA and DSC) was carried out using a TGA/DSC1 equipment (Mettler-Toledo GMBH, Spain) equipped with a microbalance (precision 0.1 μg) and with a horizontal oven, in 25–950 °C temperature range and 10 °C/min heating rate, and in atmospheric air.

Fourier-transform infrared (FT-IR) analysis was performed using a Nicolet iS20 (Thermo Scientific, Italy) equipped with a DTGS detector working in a range of 400–4000 cm^−1^ with a resolution of 8 cm^−1^.

#### 2.2.3 Synthesis and characterization of chitosan carbamate

Chitosan carbamate (CHS carbamate) was synthesized according to previous reports, with some modifications [29,30]. 0.25 g of CHS was added to 15.5 ml of 0.1 M acetic acid under magnetic stirring (molar ratio of monomer unit of CHS to acetic acid equal to 1:1). Subsequently, 1 g of NH_4_HCO_3_ was added, and the solution was left stirring overnight. Subsequently, the solution was diluted by adding 100 ml of distilled water and homogenized using an Ultra Turrax (T25 Easy Clean digital high-speed homogenizer) at 23000 rpm for 10 min.

The obtained CHS carbamate solution was lyophilized. The ^1^H NMR spectra were acquired using an NMR spectrometer (Bruker AVIII 400 MHz, Bruker Corporation, Billerica, MA, USA). To this purpose CHS carbamate was dissolved in deuterium oxide (D_2_O) and compared to pristine CHS dissolved in deuterated acetic acid. In addition, thermal (DSC and TGA) and FTIR analyses were also performed as previously described.

#### 2.2.4 Preparation of spray-dried microparticles

CHS carbamate solution was processed using a mini spray dryer (BUCHI B-190 Büchi Labortechnik AG, Germany) equipped with temperature probes (inlet temperature: 190 °C, outlet temperature: 110 °C), a vacuum filter (flow rate: 900l/h; aspirator: 100 %), a peristaltic pump (pumping speed: 25 %), and a nozzle (diameter 0.7 mm). The microparticles were collected in a reservoir and the process yield was always higher than 50 %. In addition, MMT or LDH-doped microparticles were manufactured starting from chitosan carbamate blends containing clays interaction products at 20 % w/w final concentration on dried product.

#### 2.2.5 Physico-chemical characterization of spray-dried microparticles

The microparticle morphology was assessed by SEM as previously described. In addition, the EDX spectra of the microparticles were acquired (Tescan, Mira3XMU, Brno, Czech Republic) by placing samples on metal stubs using a double-sided adhesive tape with 10 nm gold sputtering under vacuum.

Particle size distribution was assessed using a granulometer (Mastersizer 3000E, Malvern Instruments, Italy). Microparticle suspensions in isopropanol were prepared and particle diameters (D10, D50, D90, D4; 3 and Span index) were assessed. In addition, TGA/DSC, FTIR and XRPD analyses were carried out as previously described.

#### 2.2.6 *In vitro* degradation

The *in vitro* degradation was performed by incubating 10 mg/ml of microparticles in water at 37 °C. After 6 and 12 days, microparticles were centrifuged at 1000 rpm for 10 min, the supernatants were discarded, and microparticles were dried in an oven at 50 °C and weighted. The weight loss percentage was calculated as the ratio between the weight after degradation and the initial weight. Moreover, after 12 days of degradation, the morphology of microparticles was evaluated using SEM.

#### 2.2.7 Copper release

The release of Cu^2+^ over time from the clays was evaluated. 10 mg of microparticles were placed in 1 ml of distilled water at 37 °C. At prefixed times (3, 6, 12, 18 and 21 days), the microparticles were centrifuged, and supernatants (0.5 ml) were collected and replaced with fresh medium to maintain the volume constant. The samples were analyzed by means of a copper assay kit (Abcam, Prodotti Gianni Srl, Milan, Italy). At this purpose, trichloroacetic acid was added to each sample and then mixed 1:1 wt ratio with the working reagent, incubated for 5 min at room temperature and optical density was read at 359 nm using a microplate reader (FLUOstar® Omega, BMG LABTECH, Aylesbury, UK).

#### 2.2.8 Antimicrobial assay

The antimicrobial activity of microparticles was evaluated against two bacteria strains—*Staphylococcus aureus* ATCC 6538 and *Escherichia Coli* ATCC 25922. Bacteria were grown overnight in Tryptone Soya Broth (Oxoid, Basingstoke, Hampshire, UK) at 37 °C, and then centrifuged at 2000 rpm for 20 min to separate the cells from the broth and suspended in PBS. The suspension was diluted to adjust the number of cells to 10^7^–10^8^ CFU/ml (CFU = colony forming unit) and incubated at 37 °C with 500 l of 21 days release samples. Viable microbial counts were evaluated after 0, 5, and 24 h of contact and the microbiocidal effect (ME value) was calculated for each test organisms and contact times according to the following equation:

ME = logN_c_-logNd

where Nc is the number of CFU of the control microbial suspension (only microbial suspension) and Nd is the number of CFU of the microbial suspension in presence of the different types of microparticles.

#### 2.2.9 Cell biocompatibility and proliferation

Cell biocompatibility and proliferation was evaluated using normal human dermal fibroblasts from juvenile foreskin (NHDF, normal human dermal fibroblasts, PromoCell, Sigma, Milan, Italy) grown in Dulbecco's modified Eagle medium (DMEM, Sigma-Aldrich, Italy) supplemented with 10 % fetal bovine serum (FBS, Sigma, Milan, Italy), with 200 IU/ml penicillin/0.2 mg/ml streptomycin (Sigma, Milan, Italy), and kept at 37 °C in a 5 % CO_2_ with 95 % relative humidity (RH). Fibroblasts were seeded at 3.5∗10^4^ cells/well density in 96-well plate and placed in contact with the samples.

Preliminarily, the biocompatibility in the presence of pristine components was assessed. The components were dispersed in DMEM (at the same amount contained in the microparticles) and left at 37 °C for 24h. The supernatants were collected and were put in contact with the cell substrates for 24h. Then, the MTT test was performed. 100 l of the MTT solution (1 mg/ml) were dispensed into each well and incubated at 37 °C for 3h. Subsequently, the MTT solution was removed and 100 l of DMSO were added, and the absorbance was read at 570 nm with a reference wavelength of 690 nm.

Subsequently, the cell proliferation assay was performed. The microparticles were dispersed in DMEM and put in contact with the cell substrates at different concentrations (ranging from 0.1 to 2 mg/ml). NHDF grown in standard conditions (growth medium, GM) was considered as positive control. After 3 or 6 days of contact with the samples, the media were removed and 100 l AlamarBlue solution (10 % v/v in DMEM without phenol red) were added to each well. After 3 h of incubation at 37 °C, the fluorescence was read at 595 nm by means of a microplate reader with a reference wavelength of 560 nm. Moreover, the biocompatibility of the pristine materials.

#### 2.2.10 *In vitro* wound healing assay

*In vitro* wound healing assay was performed using NHDF seeded in a 24-well plate (Ibidi GmbH, Gräfelfing, Germany) consisting of two chambers with a growth area of 0.22 cm^2^ divided by a septum of 500 ± 50 μm. Fibroblasts were seeded in each chamber at 10^4^ cells density and grown for 24 h. Afterwards, the insert was removed, cell substrates were washed with PBS and incubated with 500 l of microparticles (0.1 mg/ml). Cells grown in standard conditions were used as positive control. At prefixed timepoints (0, 1, 2 and 3 days), the cells were imaged using CLSM (Leica TCS SP2, Leica Microsystems, Milan, Italy) at λex 346 nm and λem 460 nm for Hoechst 33258 and λex 501 nm and λem 523 nm for FITC-phalloidin. At this purpose, the cells were fixed with 4 % glutaraldehyde solution, and the cytoskeletons and nuclei were stained with FITC Atto 488 phalloidin (50 l at 20 μg/ml in PBS in each well, contact time 30 min) and with Hoechst 33258 (100 l at 1:10.000 dilution in PBS per each well, contact time 10 min) respectively. Wound reduction percentages were measured using ImageJ software.

#### 2.2.11 Blood-clotting assay

The blood coagulation test was performed using rat whole blood. 10 % v/v acid-citrate dextrose (ACDC, 38 mM citric acid/75 mM trisodium citrate/100 mM dextrose) was added to whole blood to avoid clotting and, prior to the experiments, the anticoagulant activity of the ACDC was inhibited by mixing with saturated CaCl_2_ solution (1:1 wt ratio). Approximately 10 mg of microparticles were mixed with 0.5 ml of incubated blood and incubated at 37 °C. After 3 min of contact, water was added and gently shaken to resuspend free red blood cells, and the absorbance of each sample was read spectrophotometrically at *λ* 542 nm using a microplate reader and the haemoglobin percentages were calculated. Additionally, clots were observed by SEM. Samples were fixed with 3 % glutaraldehyde and then dehydrated using increasing concentrations of ethanol.

#### 2.2.12 *In vivo* wound healing on a murine model

All animal tests were performed in accordance with the standard international ethical norms (European Communities Council Directive 86/609/EEC) approved by the Italian Health Ministry (D.L.116/92). The study protocol was authorised by the University of Pavia's Local Institutional Ethics Committee for the use of animals, as well as by the ISS (Istituto Superiore di Sanità). Nine male rats (Wistar 200–250 g, Envigo RMS Srl, Udine, Italy) were anaesthetized with equitensine at 3 ml/kg (39 mM pentobarbital, 256 mM chloral hydrate, 86 mM MgSO_4_, 10 % v/v ethanol, and 39.6 % v/v propylene glycol) and their backs were shaven to remove all hair. On the back of each rat, the microparticles were implanted subcutaneously in an 8 mm incision to evaluate system safety. Then the incisions were sutured using strips (Steri-Strip Suture, I). After 18 days, full thickness biopsies were obtained from the incision and the histological analysis was carried out. Intact skin biopsy was taken as a reference. Moreover, the systems effectiveness was assayed on full thickness burns. At this purpose full thickness burns (diameter of 4 mm) were made using an aluminium rod heated at 105 °C, put in contact with the back of the animal for 40 s. Three circular burns were produced and after 24h, a 4 mm diameter biopsy punch was made to remove the formed blisters and to obtain full-thickness lesions. 20 mg microparticles were applied on the lesion and wetted with saline solution. The wounds were covered with a sterile gauze and the rat back was wrapped with a surgery stretch to protect lesions. Lesions treated with saline solution were used as negative control. After 18 days, full thickness biopsies were taken in correspondence of the initial lesions and the histological analysis of the excised tissues was performed. A biopsy of intact skin was used as a positive control. The scar or residual wound area was collected, and wound tissue specimens (wound bed) were immediately immersed in fixative solution (4 % neutral buffered formaldehyde), embedded in paraffin, and sectioned at a thickness of 5 μm. The sections were stained with either hematoxylin and eosin (H&E) or picrosirius red (PSR). Stained slices were examined using a Carl Zeiss Axiophot light microscope designed for circular polarisation microscopy. The maximum length (GMTL) and thickness (GTT) of the granulation tissue were measured with the NIS-Elements software, supplied with the Nikon camera, using the Zeiss Axiophot microscope at 5× magnification.

## 3 Results and discussion

### 3.1 Physico-chemical characterization of MMT and LDH

**Fig. 1:**
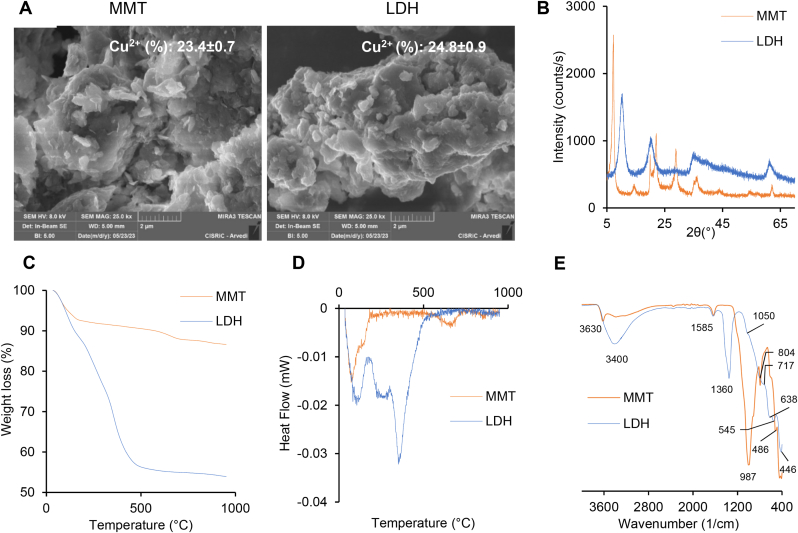
A) SEM micrographs of MMT and LDH (Cu contents are reported in the insets); B) XRPD spectra of MMT and LDH; C) TGA and D) DSC spectra of MMT and LDH; E) FTIR spectra of MMT and LDH.

Fig. 1A presents the SEM micrographs of Cu-doped montmorillonite (MMT) and layered double hydroxides (LDH). Both clays exhibit similar morphologies, characterized by a typical layered structure that includes flakes and agglomerates with poorly defined shapes. This similarity may be attributed to the raw materials used and the processes chosen: for MMT, the properties of the natural clay and the Cu^2+^ intercalation, and for LDH, the synthesis via coprecipitation in an alkaline environment and the relatively short synthesis time, which prevents crystal growth. Regardless, submicron-sized particles with a sheet-like morphology are evident in both clays. Elemental analysis (insets of Fig. 1A) confirms the presence of Cu^2+^ and its content in both MMT and LDH, ranging from 23 to 25 % w/w, consistent with the expected Cu amounts in the clays.

Fig. 1B shows the XRPD spectra, confirming the crystalline structures of the clays. The LDH diffractogram displays characteristic peaks for this type of material, with Bragg reflections indexed in the R-3m space group (hexagonal cell). Notably, three harmonic peaks at 10.2, 20.3, and 34.9 2θ, corresponding to the respective interlayer distances of 8.8, 4.4, and 2.9 Å, are detected. These values, linked to the diffraction of the (003), (006), and (009) planes, are typical of LDH with nitrate ions intercalated in the interlamellar space, expected due to the use of nitrate salts as precursors in the synthesis process [31]. The presence of broader peaks results from the synthesis method and the reduced synthesis time. Additionally, the (110) peak at 61.0 2θ (d ∼ 1.52 Å) suggests effective incorporation of Cu^2+^ within the cationic layers, consistent with the Cu^2+^ radius dimension (0.73 Å) [32,33]. The diffractogram of MMT shows the typical pattern, except for the basal reflection (001), which is shifted to slightly higher 2θ values (7.23° 2θ) compared to pristine montmorillonite (7.0° 2θ) [28]. Moreover, the interlayer space d(001) decreases from 12.6 Å for Na-MMT to 12.2 Å for Cu-MMT, likely due to the exchange between Na^+^ and Cu^2+^ in the intercalation process, as the Cu^2+^ radius (0.73 Å) is smaller than that of Na^+^ (0.95 Å), confirming the presence of Cu^2+^ in the MMT galleries (interlamellar space). Similar results are reported in the literature [34].

Fig. 1C and D illustrate the thermal behaviour of MMT and LDH. The TGA profiles suggest that both Cu^2+^-clays exhibit weight loss as temperature increases. MMT shows an initial weight loss of about 8 % (w/w), corresponding to the loss of water of hydration. A typical dehydroxylation phase of clay minerals starts at approximately 600 °C and offsets at 720 °C, representing a weight loss of 3 % (w/w). From 800 °C onwards, a slight weight loss (less than 1 %) is observable, probably related to the decomposition of some impurities. On the other hand, LDH shows a four-stage weight loss. Initially, there is a weight loss around 100 °C related to the loss of hydration water adsorbed on the cationic layers. Subsequently, two other stages are identifiable up to 400 °C, mainly attributable to the dehydroxylation of the octahedral cationic layers, leading to the elimination of water molecules, and the removal of nitrate ions from the interlamellar spaces [35]. Finally, a slight weight loss (about 5 %) is observed, likely due to the elimination of some impurities derived from the synthesis. The DSC profiles corroborate the TGA results.

Fig. 1E displays the FTIR spectra of both montmorillonite and layered double hydroxides. The MMT spectrum is characterized by several distinct bands: a band at 3630 cm^−1^ corresponding to Al-OH-Al, Al-OH-Mg, and Mg-OH-Mg vibrations, a broad band around 3400 cm^−1^ due to O-H stretching, a band at 1585 cm^−1^ related to H-O-H bending. In addition, typical bands of MMT at 987 cm^−1^ (Si-O stretching), and at 486 cm^−1^ (Si-O bending) are present. The presence of intercalated Cu^2+^ is confirmed by a slight increase in the wavenumber of the O-H stretching band compared to pristine montmorillonite, likely related to Cu^2+^ solvation [28,36].

Analogously, the LDH spectrum shows the characteristic bands of the layered double hydroxides structure. A broad band is observed in the region between 3750 and 2700 cm^−1^, related to the bridging of the bond between H_2_O and the interlayer anion, the H-bonded interlayer H_2_O surrounding the interlayer anion, and the metal-OH stretching. In addition, a weak band is observed at around 1630 cm^−1^ due to the bending of interlayer H_2_O and a more intense signal is detected around 1360 cm^−1^ due to the *ν*_3_ anti-symmetric stretching mode of NO_3_^−^. Moreover, at 804 cm^−1^, a weak out-of-plane symmetric deformation (*ν*_2_) of NO_3_ is present. On the lower end of the spectrum, several bands related to the presence of the cations can be observed: 1050 cm^−1^ (Al-OH deformation), 717 cm^−1^ (Al–OH translation or Cu-O stretching vibrations), 638 cm^−1^ (Mg–OH translation), 545 cm^−1^ (Al–OH translation), and 446 cm^−1^ (HO–metal–OH deformation) [37,38].

### 3.2 Physico-chemical characterization of chitosan carbamate

**Fig. 2:**
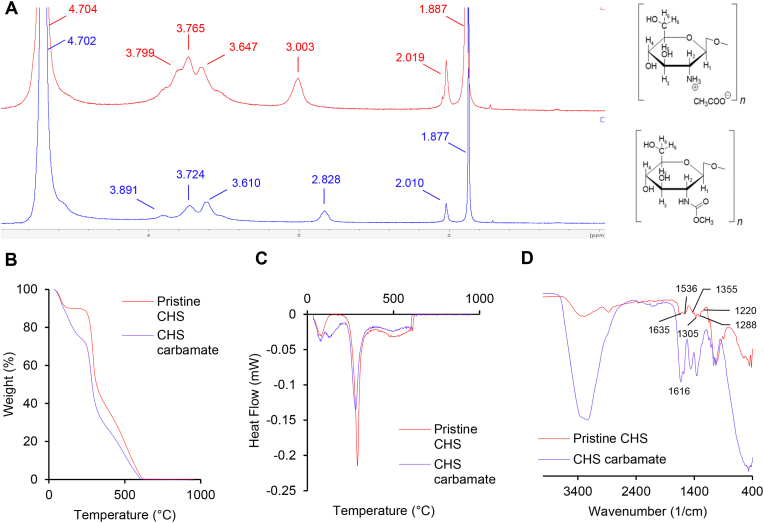
A) ^1^H NMR of pristine (in red) and carbamate (in blue); B) and C) TGA and DSC spectra of pristine (in red) and carbamate (in blue); D) FTIR spectra of pristine (in red) and carbamate (in blue). (For interpretation of the references to color in this figure legend, the reader is referred to the Web version of this article.)

Fig. 2A compares the ^1^H NMR spectra of chitosan acetate (pristine CHS) and chitosan carbamate (CHS carbamate), synthesized from CHS. The signals detected at 3.0 ppm and in the range of 3.6–3.8 ppm correspond to H-2 and H-3-6 of CHS, respectively. The expected H-1 peak at 4.57 ppm is not visible due to overlapping with the solvent signal. Additionally, CH_3_ signals of the acetylic group and acetate salt of CHS are observed at 2.0 ppm and 1.88 ppm, respectively. The signal of free acetic acid is not detected, likely due to the total dissociation of acetic acid as the acetate counterion of CHS, attributed to the 1:1 M ratio between the CHS glucosamine moiety and acetic acid.

For CHS carbamate, ammonium bicarbonate is added in a 4-fold molar ratio relative to CHS, resulting in variations in the observed signals. Specifically, the CH_3_ signal of CHS acetate is replaced by a different peak at a lower ppm, presumably due to the formation of ammonium acetate from ammonium bicarbonate during the synthesis of CHS carbamate. Additionally, the H-2 signal shifts to 2.8 ppm, consistent with the presence of the carbamate function, as reported in the literature [30]. These NMR spectral features indicate that CHS is completely converted into the carbamate form.

Fig. 2B and C shows the thermal analyses (TGA and DSC, respectively) comparing the thermal stability of chitosan acetate (pristine CHS) and chitosan carbamate (CHS carbamate).

The thermogram of pristine CHS shows three degradation stages. An initial degradation occurs at approximately 40–100 °C with a weight loss of 10 % corresponding to water evaporation. A second degradation phase occurs from 250 °C to 290 °C, with a weight loss of about 40 %. Lastly, at about 640 °C, the complete carbonization of CHS is observed. On the other hand, CHS carbamate shows a four-stage degradation. The first phase, between 40 and 100 °C, corresponds to the evaporation of water, followed by a second degradation in the range between 120 and 220 °C, likely due to the decomposition of ammonium bicarbonate residues. The third decomposition phase occurs in the range of 250–320 °C with a weight loss of approximately 37 %. Finally, a complete carbonization at a temperature of approximately 625 °C is detected.

In agreement with TGA, the DSC thermogram of CHS shows three broad endothermic peaks at around 60 °C, 290 °C, and 490 °C, related to water loss, CHS molecular arrangement, and CHS carbonization, respectively [33]. In contrast, the DSC thermogram of CHS carbamate reveals four broad endothermic peaks at around 60 °C, 140 °C, 290 °C, and 490 °C, related to water loss, decomposition of ammonium bicarbonate residues, CHS molecular arrangement, and CHS carbonization, respectively. These results indicate that the structure of chitosan chains changes due to derivatization with carbamate moieties. Additionally, CHS carbamate exhibits faster degradation at lower temperatures compared to pristine CHS, likely due to weaker intermolecular hydrogen bonds among the polymer chains.

Fig. 2D shows the FTIR spectra of pristine CHS and CHS carbamate. The spectrum of pristine CHS aligns with the specific peak assignments reported in the literature. The characteristic bands are observed at 1635 cm^−1^ (amide I band), 1536 cm^−1^ (amide II band), 1220 cm^−1^ (amide III band), 1355 and 1305 cm^−1^ (O–H and C–H vibrations in the ring), 1288 cm^−1^ (C–H bond vibrations of the methyl group in the amide group) [29].

The FTIR spectrum of CHS carbamate shows notable differences compared to pristine CHS: the amide I band at 1635 cm^−1^ is absent due to the consumption of NH_2_ groups in the reaction with ammonium bicarbonate, while a new absorption peak appears at 1616 cm^−1^, related to the stretching vibration of the carbonyl (C=O) of -NHCOO- [29,30]. These spectral features confirm the successful conversion of CHS into the carbamate form.

### 3.3 Preparation of spray-dried microparticles

Following the synthesis of MMT, LDH, and CHS carbamate, three different polymeric blends were prepared: one based solely on CHS carbamate, one doped with MMT, and one doped with LDH. These blends were then subjected to a spray drying process, as shown in the schematic image of the work reported in Fig. S1. The process, conducted at an inlet temperature of 190 °C, induces the thermal decomposition of excess ammonium bicarbonate and the CHS carbamate to the CHS free base, releasing carbon dioxide as a side product. This results in the formation of insoluble microparticles in an aqueous environment, avoiding the use of chemical crosslinking agents.

**Fig. 3:**
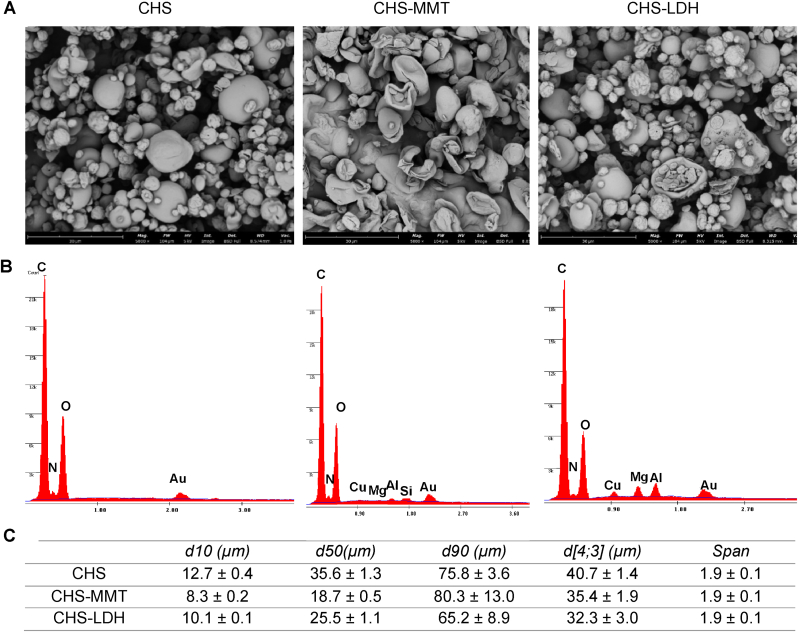
A) SEM images of spray-dried microparticles; B) EDX spectra of spray-dried microparticles; C) statistical particle parameters of spray-dried microparticles (mean values ± s.d; n = 3). Anova One-Way, Scheffé test, p < 0.05: d[4; 3], CHS vs CHS-MMT, CHS vs CHS-LDH, CHS-MMT vs CHS-LDH.

Fig. 3A presents SEM micrographs of the microparticles obtained via the spray drying process. The microparticles exhibit different morphologies depending on their composition: CHS-based microparticles appear heterogeneous with a smooth surface and predominantly spherical shape while microparticles doped with clays (MMT or LDH) show different morphologies, characterized by wrinkled surfaces with concavities and porosity. In addition, the EDX spectra confirm the presence of the characteristic elements of the clays in the final structure, in particular the cations related to the structure of MMT and LDH.

Fig. 3B displays the particle size distribution data. All microparticles feature a narrow particle size distribution, with a Span index lower than 2. The mean particle size (d[4; 3]) ranges between 32 and 40 μm. Interestingly, clay doping results in smaller microparticles compared to undoped ones. This size reduction could be attributed to the interaction between CHS and clays, involving polymer chains intercalating into the clay galleries during the preparation of the polymeric blend.

These findings suggest that the incorporation of clays into the CHS matrix influences both the morphology and size of the resulting microparticles, potentially impacting their functional properties for wound healing applications.

### 3.4 Physico-chemical characterization of spray-dried microparticles

**Fig. 4:**
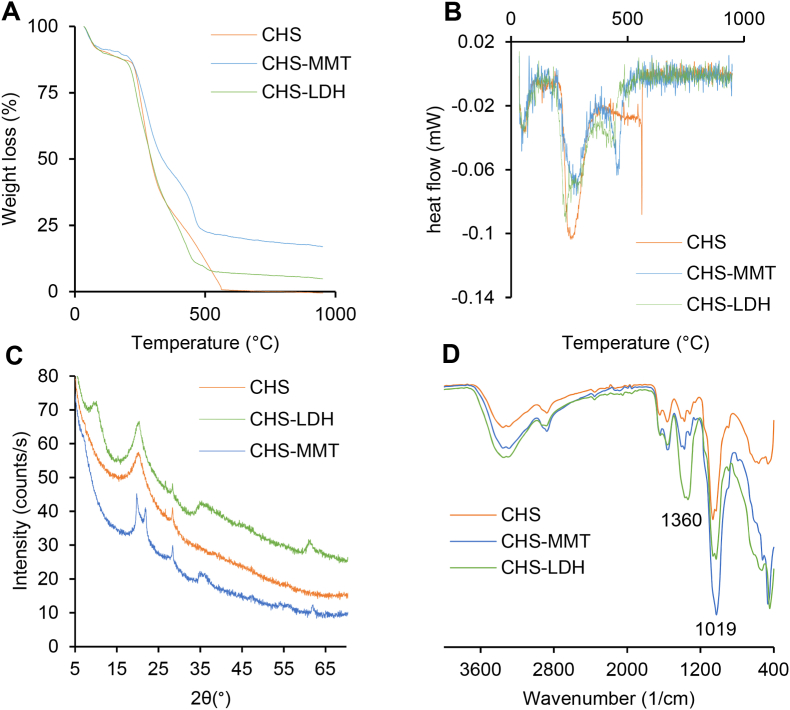
A) and B) TGA and DSC spectra of spray-dried microparticles; C) XRPD spectra of spray-dried microparticles; D) FTIR spectra of spray-dried microparticles.

The results of the thermal analysis of the spray-dried undoped microparticles and those doped with MMT or LDH are presented in Fig. 4A and B. All formulations exhibit a three-stage weight loss profile: an initial stage at 100 °C characterized by a weight loss, amounting to about 10 % of the total mass, is attributed to water evaporation. A second stage at 200–350 °C is likely due to the degradation of chitosan (CHS), and a third stage at 350–900 °C, where the microparticles show different behaviors depending on the doping. The undoped microparticles completely degrade at around 580 °C while doped microparticles show final residues, confirming the presence of the clays. The DSC profiles (Fig. 4B) are consistent with the TGA analysis, reinforcing these findings.

Fig. 4C shows the XRPD spectra of the spray-dried microparticles. The undoped microparticles display an intense diffraction peak at around 20° 2θ and two smaller peaks around 26° and 28° 2θ, associated with the (001) and (100) planes of the monoclinic system. Additionally, a weak diffraction peak at around 10° 2θ indicates the paracrystalline structure of CHS [39]. CHS-LDH microparticles display a similar pattern to that of the undoped microparticles. However, compared to the pristine LDH (Fig. 1B), the peaks (003) and (110) related to LDH are clearly detectable at 10.2° and 61.0° 2θ. Their interlayer distances remain unaltered after inclusion in the microparticles. The peak (006), previously observed around 20° 2θ, is completely obscured by the paracrystalline CHS signal.

CHS-MMT microparticles are characterized by the presence of MMT that is identifiable by its typical pattern. Compared to the MMT diffractogram (Fig. 1B), the basal reflection d001 of CHS-MMT microparticles shifts from 7.23° 2θ to approximately 6.93° 2θ. This shift, corresponding to an increase of 0.3 Å in the interlayer space, suggests the co-intercalation of CHS into the MMT layers alongside Cu^2+^, likely forming a monolayer between the silicate layers.

These results indicate that doping with MMT or LDH alters the thermal stability and structural characteristics of the chitosan-based microparticles. Such incorporation of these functional inorganics into polymeric systems leads to composite materials that not only resemble more effectively ECM but also should confer the desired mechanical strength, bioactivity, and biocompatibility that could support tissue regeneration. Various works reported the doping of different functional inorganics into polymeric networks for tissue engineering purposes [[40], [41], [42]]. The addition of these inorganics affects the thermal stability and structural properties of the polymeric network, allowing a degradation rate of the polymeric matrix matching the needs of tissue regeneration. These thermal and structural changes can also determine prolonged release of therapeutic agents, enhancing wound healing.

Fig. 4D presents the FTIR spectra for CHS microparticles and clay-doped microparticles. The undoped microparticles exhibit an identical pattern to pristine CHS, indicating the successful conversion of CHS carbamate to CHS during the spray drying process. Regardless of the type of clay used, all the microparticle spectra are dominated by the presence of CHS. However, at low wavenumbers, CHS-MMT displays a characteristic silicate band at 1018 cm^−1^, while CHS-LDH shows a typical band around 1360 cm^−1^, which is associated with NO_3_^−^ ions present in the interlayer space [28,37].

### 3.5 *In vitro* degradation

**Fig. 5:**
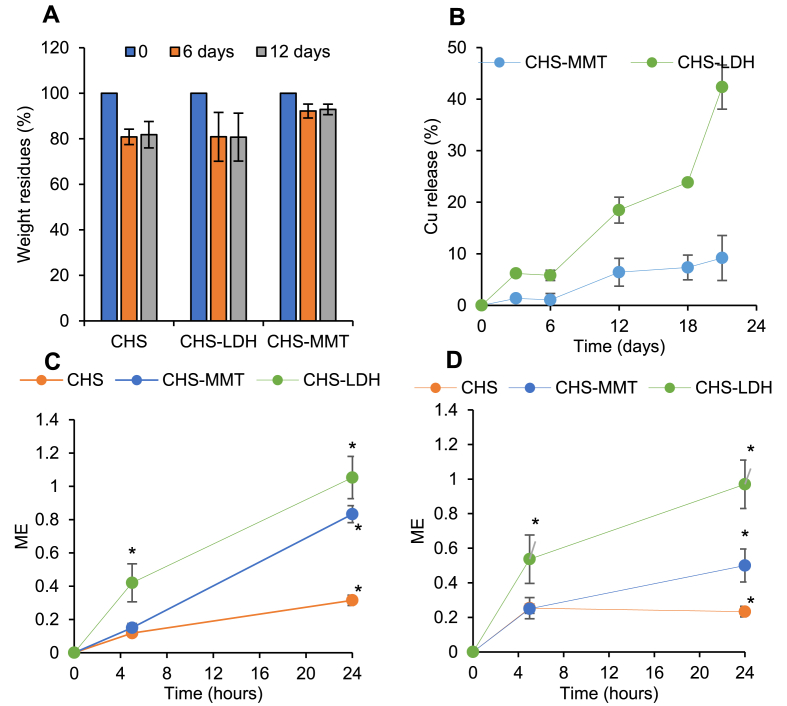
A) Weight residues of spray-dried microspheres after 6 and 12 days of incubation in water at 37 °C; B) copper released from the microparticles after 3, 6, 12, 18, and 24 days (mean values ± s.d; n = 3), C) and D) time killing curves (ME values vs time) for microparticles against *E. coli* ATCC 8739 (left panel) and *S. aureus* ATCC 29213 (right panel).

Since behavior in an aqueous environment is crucial for scaffolds and formulations in wound healing, the microparticles developed in this work have been subjected to hydration. Their weight loss and degradation have been monitored over time up to 12 days at 37 °C (Fig. 5A). Wound hydration is a key factor in hemostasis, and a balance between exudate absorption and wound dehydration should be maintained to support the granulation phase of the healing process.

The microparticles exhibit slow degradation over time, with less than 20 % weight loss observed. Specifically, CHS and CHS-LDH microparticles retain their structure with approximately 80 % residue, while CHS-MMT microparticles are more resistant to degradation, maintaining a residue of 92 %. The clay doping, particularly with MMT, appears to reduce the degradation process of the microparticles. This reduction is likely due to the clay interacting with the polymer matrix, acting as an assembly point, and decreasing the mobility of polymer chains. This also suggests the stability of the microparticles in an aqueous environment. However, it should be noted that the chitosan matrix *in vivo* is susceptible to lysozyme, an enzyme secreted by white blood cells that cleaves β-(1,4)-glycosidic bonds, which are recruited to the lesion bed during the inflammatory phase of wound healing [43].

### 3.6 Cu^2+^ release and antimicrobial properties

Fig. 5B shows the percentage of Cu^2+^ released over time from CHS-MMT and CHS-LDH microparticles. Regardless of the type of clay, Cu^2+^ is released slowly in a controlled and sustained manner. After 21 days, 42 % of Cu^2+^ is released from CHS-LDH microparticles, while CHS-MMT microparticles release 9 % of Cu^2+^. The slower release from CHS-MMT microparticles confirms that MMT is more stable than LDH, which is consistent with thermal analysis investigations. The release profiles align with *in vitro* degradation results; higher degradation correlates with higher release due to matrix breakdown. Similar findings were reported by Kottegoda et al. [44], who showed that the relase of urea was slower for MMT than that from LDH urea was released from MMT more slowly compared to urea from LDH. In addition to the type of clay, Cu^2+^ release is also controlled by the polymeric matrix, suggesting that the chitosan network hinders ion diffusion. Copper plays an important role in wound healing by stimulating neovascularization, increasing collagen production, and mostly providing high antimicrobial activity, which is important in preventing infection at the wound site [45,46]. However, a valid concern is the potential toxicity due to excessive release of copper. Transition metals, like copper, can induce the formation of ROS, resulting in cell damage [47,48]. At this purpose, in this work Cu^2+^ ions were incorporated into clay mineral-doped microparticles to mitigate such risks. The results showed that Cu^2+^ ions were slowly released, avoiding toxicity which could be caused by burst effect.

Microbial contamination, infection, and biofilm formation are major challenges in chronic wounds, causing healing delays, tissue necrosis, and infection spread [49]. The activity of the microparticles against two representative bacterial strains, *E. coli* (ATCC 8739, Gram-negative) and *S. aureus* (ATCC 29213, Gram-positive), has been tested (Fig. 5C and D). The microparticles exhibit good antimicrobial properties up to 24h, with an onset after 5h of contact. Overall, they are more effective against Gram-negative bacteria than Gram-positive ones. CHS-LDH microparticles seem more effective *in vitro*, likely due to the greater Cu^2+^ release. Cu^2+^ exerts its antibacterial properties by interacting with thiol and amine groups in cell membrane proteins, leading to bacterial death. Additionally, Cu^2+^ can bind DNA molecules, disrupting the helical structure [50]. The greater sensitivity of *E. coli* to clay-doped microparticles compared to *S. aureus* may be due to the higher affinity of Cu^2+^ for amine and carboxyl groups, which are abundant on the cell surface [51]. These findings confirm the inherent properties of copper and its use in commercial products (MedCu wound dressings) due to its potent antimicrobial efficacy [52].

However, undoped microparticles also show mild antibacterial activity, likely due to the intrinsic properties of chitosan. Chitosan acts as a bacteriostatic agent through electrostatic interactions between its amino groups and negatively charged cell membranes, causing permeability alteration and membrane disruption [53].

### 3.7 Cell proliferation and *in vitro* wound healing assay

The biocompatibility assessment of the microparticle components after 24 h of growth is shown in Fig. S2. Since a 30 % decrease in viability of Normal Human Dermal Fibroblasts (NHDF) is within the biocompatibility range according to the international standard ISO 10993-5, all components were characterized by good biocompatibility.

**Fig. 6:**
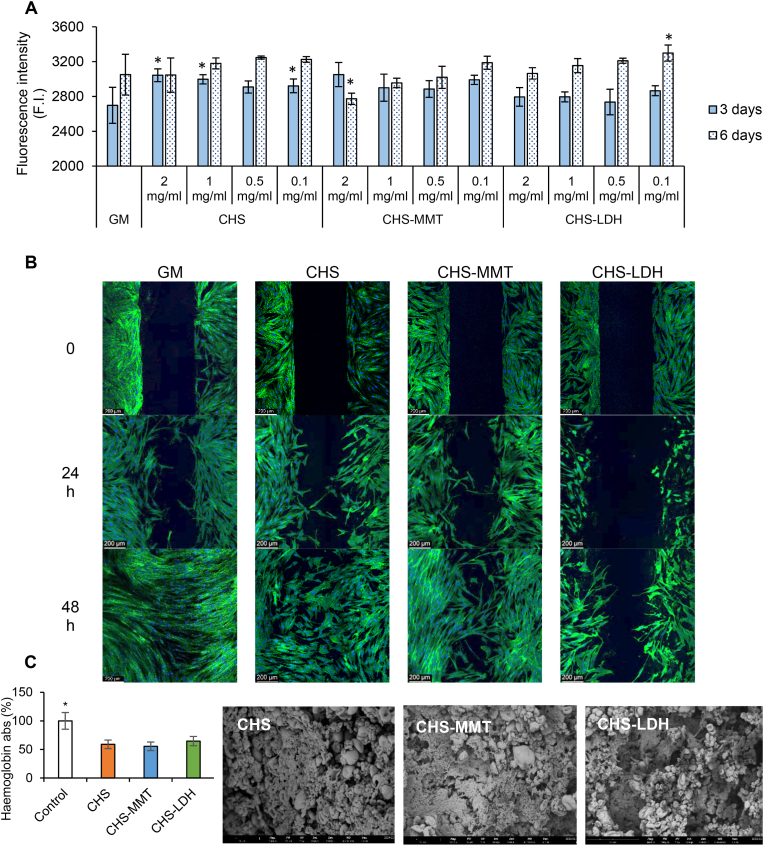
A) Viability of fibroblasts after 3 days and 6 days of contact with spray dried microparticles in comparison to the positive control GM (growth medium, as standard growth conditions) ((mean values ± s.d; n = 3). Values ∗ are statistically significant (p < 0.05) compared with control. B) CLSM images of fibroblasts during the wound healing assay. C) % Haemoglobin absorbance profiles after 3 min of contact with microparticles (mean values ± s.d; n = 3) and SEM images of the mixture of microparticles and the whole. Values ∗ indicate significant differences (p < 0.05).

Moreover, the cell proliferation of the microparticles towards NHDF was compared to the viability of cells grown under standard conditions (Fig. 6A). It is evident that NHDF are actively proliferating during the assay, as the 6-day viability is higher than the 3-day viability when the microparticles concentration is ≤ 1 mg/ml. Over a longer period, CHS-LDH microparticles demonstrate the best performance.

Moreover, the ability of the microparticles to enhance wound healing was investigated *in vitro* using a wound healing assay (Fig. 6B). At time zero, the CLSM micrographs of NHDF substrates show 500 μm gaps bordered by confluent cells, mimicking wounds. After 24 h, NHDF begin to close the gaps and exhibit the morphology typical of migratory cells, forming contact points across the gaps to bridge the two opposite borders. After 48 h of growth, NHDF populate the gap area, reaching confluence in most samples. The percentage of wound area reduction, reported in Fig. S3, suggests that both CHS (undoped microparticles) and CHS-MMT show similar behaviour compared to the control (cells grown under standard conditions). However, a worse performance is observed for CHS-LDH microparticles. This is likely due to the higher Cu^2+^ release, which slightly impairs the proliferation process in the short term, although the cells are able to recover over a longer period. Regardless of the type of microparticles, NHDF maintain their typical fusiform morphology with elongated cytoskeletons.

### 3.8 Blood-clotting assay

The microparticles are also characterized by good hemostatic properties (Fig. 6C). All the microparticles, regardless of their composition, are able to reduce free hemoglobin by 55–66 % compared to the control, indicating faster clot formation and suggesting good hemostatic properties. Chitosan is conceivably the major responsible for the hemostatic activity, as it is known to promote the aggregation of negatively charged erythrocytes and thrombocytes to form blood clots, thereby stopping bleeding [54].

However, both montmorillonite and layered double hydroxides may also contribute to hemostasis. Previous studies have reported that montmorillonite is an excellent hemostatic agent due to its absorbent properties and anionic surface charge, which can activate blood coagulation [55]. Similarly, the positively charged surfaces of layered double hydroxyde, resulting from magnesium and aluminum hydroxide layers, enhance hemostasis by promoting tissue and red blood cell adhesion [56].

SEM images (Fig. 6C) reveal red blood cells and microparticles entrapped in a fibrin network, supporting the quantitative data of free hemoglobin reduction.

These evaluations confirm that chitosan and clays proved to maintain their inherent properties despite the actual composition and process to obtain microparticles. In particular, chitosan and clays are the main components of hemostatic medical devices (chitosan: Hemcon®, Chitoflex®, clays: Woundstat™) [[57], [58], [59], [60]].

### 3.9 *In vivo* wound healing on a murine model

**Fig. 7:**
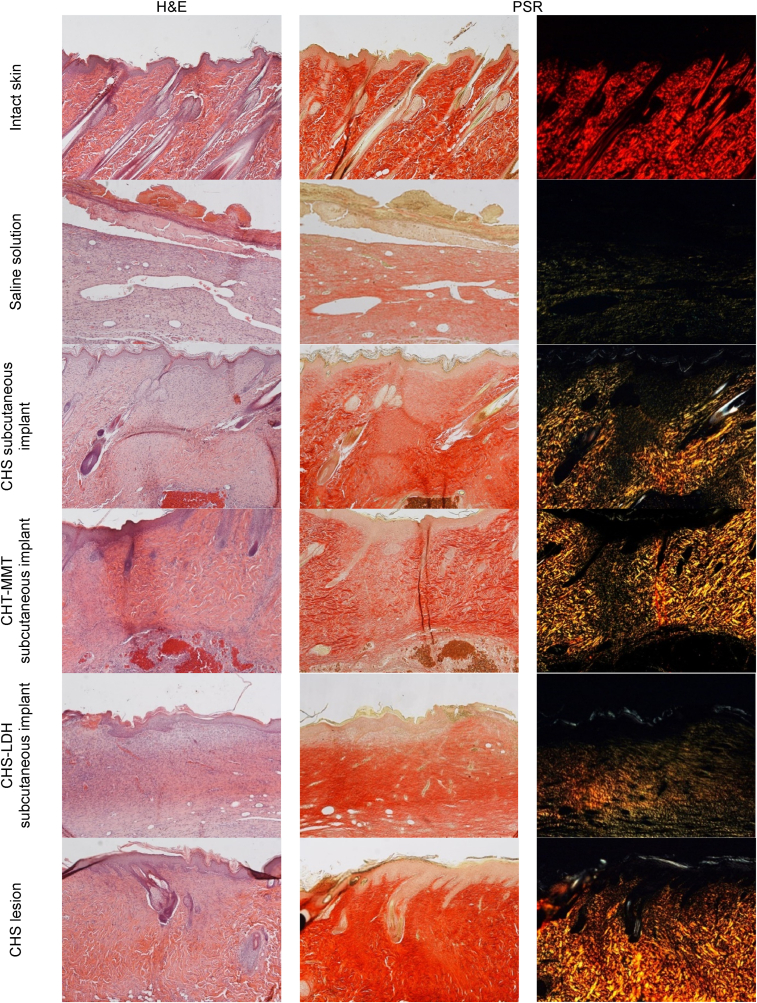

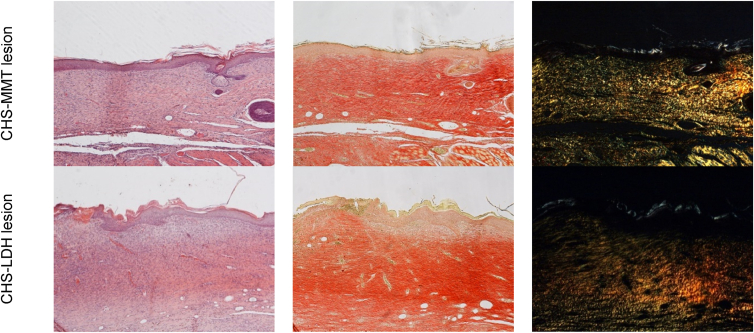
) H&E (left panel) and PSR (central panel - with bright-field images; right panel - with polarized light) sections of intact skin, lesions treated with saline solution as negative control and with spray-dried microparticles after 17 days of treatment. Original magnification: 5X. Each micrograph frame has a width of 1780 μm.

The preclinical study on safety suggests that all the formulations are biocompatible in a murine model (Fig. 7, "implant"). H&E staining does not show any signs of inflammation or foreign body reaction. The damaged tissues caused by microparticle implants are fully recovered, with completely and correctly reformed epidermal layers. In particular, CHS microparticles promote the reconstitution of the underlying connective tissue and the reorganization of the papillary and reticular dermis areas, which are characterized by thin and thick collagen bundles and neo-formed skin appendages. CHS-MMT not only enables complete organization of collagen in the papillary and reticular areas but also supports the presence of hair follicles and sebaceous glands. In contrast, CHS-LDH leaves residues of granulation tissue, which is consistent with the *in vitro* wound healing evaluation. PSR staining confirms these findings, showing the collagen bundles already remodeled and arranged as in intact skin for CHS and CHS-MMT (red signal), whereas CHS-LDH results in a lack of collagen bundles (yellow-green signal).

Moreover, the efficacy of the microparticles to promote tissue regeneration was evaluated (Fig. 7, "lesion"). The CHS-treated wounds are completely re-epithelialized, with a well-keratinized epidermis organized in multiple layers. The dermis is remodeled in its papillary and reticular parts, with collagen bundles arranged similarly to those in intact skin, and glands and hair follicles are reforming. Picrosirius red staining shows many bundles of organized and oriented collagen, yellow-red in color, like those of intact skin.

10.13039/100014337Furthermore, the preclinical model supports the effectiveness of the Cu^2+^ clay doped microparticles. In H&E analysis, CHS-MMT and CHS-LDH microparticles show similar activity on full-thickness lesions. The healing process enhanced by the microparticles allows the restoration of a multi-layered epidermis with evident keratinization. Additionally, the dermal papillae are present, as well as skin appendages. Several blood vessels are evident in the dermis, suggesting neo-angiogenesis, although the collagen in the dermal layer is immature and organized in thin bundles. PSR staining aligns with H&E staining, highlighting newly formed collagen (green-yellow signal) with only a few bundles (orange signal). To support this, wound closure (Fig. S4) after 18 days of treatment with a complete or almost complete epidermidis regeneration is assessable [61]. The positive impact of all the scaffolds on wound regeneration is also visible from the statistically significant reduction of the granulation tissue maximal length (GTML) and thickness (GTT) against the control, as shown in Fig. S5.

In contrast, the negative control (saline solution) is not re-epithelialized, and the lesion surface is covered by acidophilic necrotic material, while the underlying dermis is infiltrated by inflammatory cells and several dilated capillaries are evident. Dermal papillae or skin appendages are not observed.

Overall, wounds treated with all types of microparticles show signs of the proliferative phase of healing, demonstrating that the developed systems enhance the regeneration process. Additionally, no signs of fibrosis, inflammation, or residues of microparticles are present in any case, indicating the safety and complete *in vivo* degradation of the microparticles.

## 4 Conclusions

Chitosan-based microparticles doped with inorganic clay minerals were successfully designed and developed for skin tissue regeneration. The scaffolds were prepared via the spray drying process following the synthesis of a chitosan derivative, chitosan carbamate, which is soluble in a pH-neutral environment. This enabled the incorporation of two types of inorganic clay minerals into the microparticles: naturally occurring montmorillonite and synthetic layered double hydroxide. Both clays were tailored to include copper in their structure, enhancing the antimicrobial properties of the final product. All solutions processed through spray drying resulted in the formation of water-insoluble microparticles with a narrow particle size distribution.

Chitosan, clays and copper ions proved *in vitro* to maintain their inherent antimicrobial and hemostatic properties. Among the formulations, CHS-LDH microparticles demonstrated the highest antimicrobial activity, particularly effective against Gram-negative bacteria. However, the pronounced antimicrobial effect of CHS-LDH caused a slight delay in *in vitro* wound healing. Nevertheless, preclinical *in vivo* investigations confirmed the safety and efficacy of both the clay-doped microparticles and the undoped systems.

Overall, the developed chitosan microparticles doped with MMT or LDH not only enhance the pro-healing properties through controlled copper release, hemostatic and antimicrobial activities, but also act as highly effective scaffolds. These are expected to provide structural support for the growth of new tissue, integrating with the surrounding biological environment and acting as a temporary matrix that guides cell proliferation and tissue repair. As the wound heals, these scaffolds should gradually degrade and allow the newly formed tissue. Moreover, the presence of MMT or LDH provides structural reinforcement to the chitosan matrix, promoting cellular proliferation and supporting the natural tissue repair process.

Clay minerals proved to be novel enhancers of the antimicrobial activity due to the inclusion of copper as a bioactive ion. This allowed the development of a drug-free system as an attractive device to fight antimicrobial resistance. Furthermore, to the best of our knowledge, this is the first study that compares the effects of natural and synthetic clay minerals included in a scaffold, demonstrating how their peculiar properties affect the system. The processes developed are promising to have a fast scalability with high industrial application potential. The combination of these multifunctional materials provides mechanical support, antimicrobial activity, degradability and biocompatibility in a single system capable of promoting wound healing by mimicking the ECM, while preventing infections.

## CRediT authorship contribution statement

**Marco Ruggeri:** Writing – review & editing, Writing – original draft, Supervision, Methodology, Investigation, Formal analysis, Data curation, Conceptualization. **Cristian Nomicisio:** Writing – original draft, Methodology, Investigation, Formal analysis, Data curation. **Christine Taviot-Guého:** Resources, Conceptualization. **Barbara Vigani:** Visualization, Validation. **Cinzia Boselli:** Investigation. **Pietro Grisoli:** Investigation. **Antonia Icaro Cornaglia:** Investigation. **Eleonora Bianchi:** Visualization. **César Viseras:** Validation, Resources. **Silvia Rossi:** Visualization, Validation. **Giuseppina Sandri:** Writing – review & editing, Supervision, Resources, Project administration, Funding acquisition, Conceptualization.

## Declaration of competing interest

The authors declare the following financial interests/personal relationships which may be considered as potential competing interests: Giuseppina Sandri reports financial support was provided by The National Recovery and Resilience Plan (NRRP). If there are other authors, they declare that they have no known competing financial interests or personal relationships that could have appeared to influence the work reported in this paper.

## Data Availability

Data will be made available on request.
