# Supplementary material for: Smart copper-doped clays in biomimetic microparticles for wound healing and infection control
Source: Mater Today Bio. 2024 Oct 9;29:101292. doi: 10.1016/j.mtbio.2024.101292 (PMC11525154; doi:10.1016/j.mtbio.2024.101292)
Supplement: Multimedia component 1 [file mmc1.docx]

Supplementary Information

Smart copper-doped clays in biomimetic microparticles for wound healing and infection control

Marco Ruggeri^1^, Cristian Nomicisio^1^, Christine Taviot-Guého^2^, Barbara Vigani^1^, Cinzia Boselli^1^, Pietro Grisoli^1^, Antonia Icaro Cornaglia^3^, Eleonora Bianchi^1^, César Viseras^4^, Silvia Rossi^1^, Giuseppina Sandri^1^

^1^ Department of Drug Sciences, University of Pavia, Viale Taramelli 12, 27100 Pavia, Italy;

^2^ Institut de Chimie de Clermont-Ferrand, Université Clermont-Auvergne, UMR CNRS 6296, 24 av Blaise Pascal, 63171 Aubière, France;

^3^ Department of Public Health, Experimental and Forensic Medicine, University of Pavia, via Forlanini 2, 27100, Pavia, Italy;

^4^ Department of Pharmacy and Pharmaceutical Technology, Faculty of Pharmacy, University of Granada, Campus of Cartuja, 18071 Granada, Spain.

Corresponding author: [giuseppina.sandri@unipv.it](mailto:giuseppina.sandri@unipv.it)


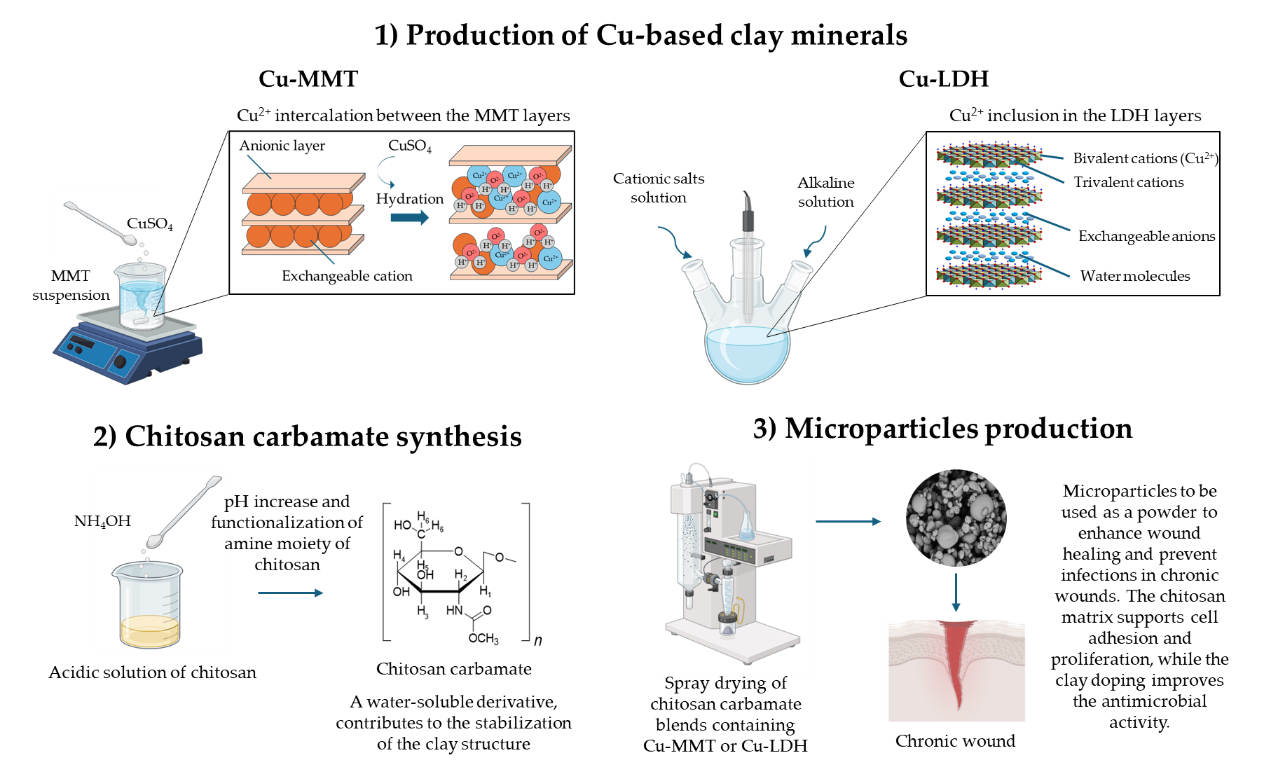


Figure S1: schematic image of the project.

Figure S2: cell viability percentages of NHDF after 24h of contact with the pristine materials (mean values ± sd; n=3).

Figure S3: Wound area reduction (%) obtained from confocal images of fibroblasts growing during the wound healing assay: * indicates significative differences (mean values ± sd, n = 3).

| **B**  **A** | | | | |
| --- | --- | --- | --- | --- |
|  | Day 0 | Day 7 | Day 14 | Day 18 |
| NaCl 0.9% | 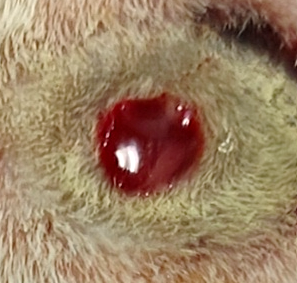 | 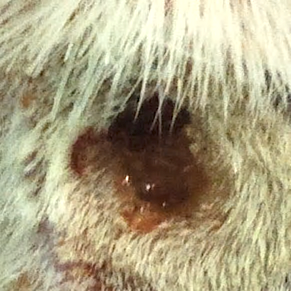 | 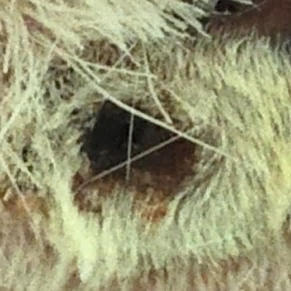 | 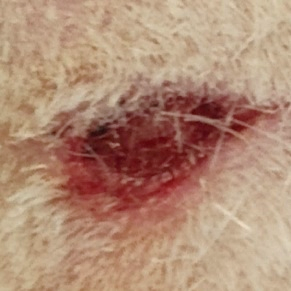 |
| CHS | 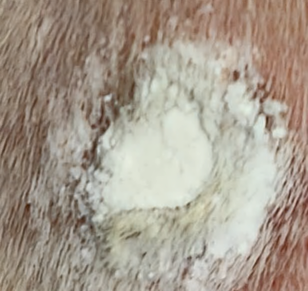 | 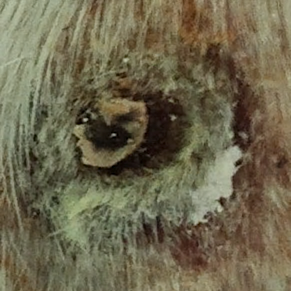 | 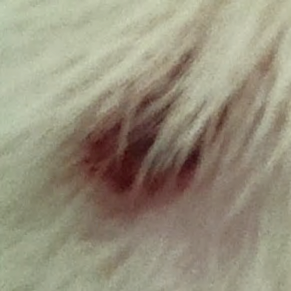 | 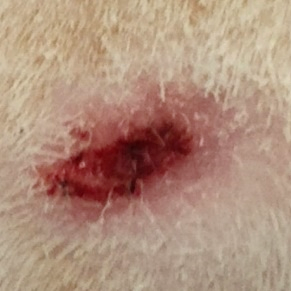 |
| CHS-MMT | 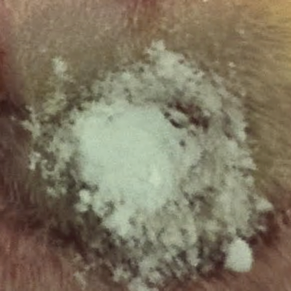 | 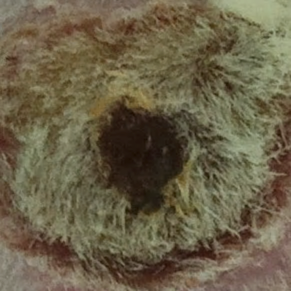 | 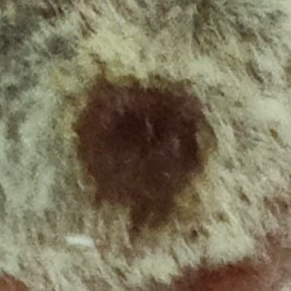 | 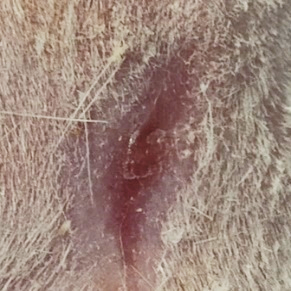 |
| CHS-LDH | 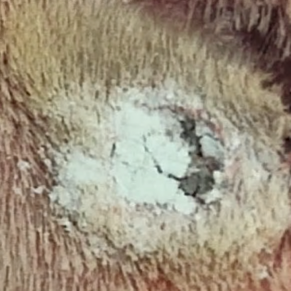 | 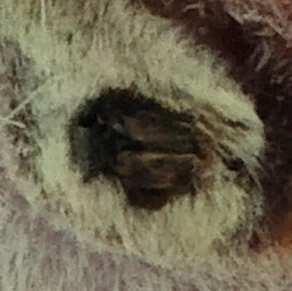 | 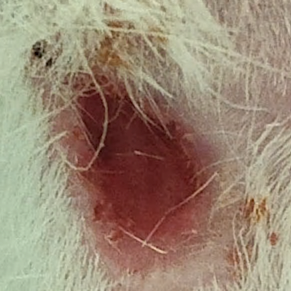 | 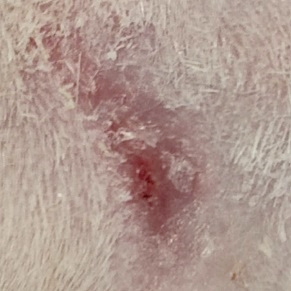  1 cm |

Figure S4: A) Lesion area vs. time obtained during the treatments using microparticles and saline solution (negative control) in an in vivo murine burn/excisional model (mean values ±sd; n=3). B) Photographs of the progression of wound healing over time.

Figure S5: Granulation tissue maximal length (GTML) and thickness (GTT) following the treatment with microparticles and saline solution (negative control): * indicates significative differences (mean values ± sd, n = 3).
